# The effectiveness of nurses’ psychosocial interventions for sensory deprivation in intensive care patients: a systematic review and meta-analysis

**DOI:** 10.1007/s11845-024-03735-0

**Published:** 2024-06-25

**Authors:** Sevda Uzun

**Affiliations:** Department of Psychiatric Nursing, Gümüşhane University Faculty of Health Sciences, Gümüşhane, Turkey

**Keywords:** Critical care, Nurse, Sensory deprivation, Systematic review and meta-analysis

## Abstract

**Objective:**

This systematic review and meta-analysis aimed to assess the effectiveness of nurses’ psychosocial interventions for addressing sensory deprivation in intensive care units (ICUs).

**Materials and methods:**

A comprehensive search of PubMed, Web of Science, EBSCOhost, Google Scholar, CİNAHL, Embase, Cochrane Library, and YÖK Thesis Center databases was conducted from August 2023 to May 2024, without any temporal restrictions. In addition, a physical search was made in the university library for grey literature.

**Results:**

The study revealed that nurses’ psychosocial interventions significantly improved patients’ level of consciousness (SMD = 1.042, %95 CI = 0.716 to 1.369; *Z* = 6.25; *p* < .05) and sleep quality in ICUs (SMD=1.21, 95% CI= 0.232 to 1.810; *Z* = 2.49; *p* < .05). The effectiveness of psychosocial interventions varied based on the type of intervention, patient age, ICU type, patient group, and intervention duration. Notably, auditory stimuli and aromatherapy demonstrated particularly high effect sizes, significantly enhancing patients’ levels of consciousness and sleep quality.

**Conclusion:**

In conclusion, psychosocial interventions aimed at reducing sensory deprivation in intensive care units exert beneficial effects on individuals, notably enhancing their level of consciousness and improving sleep quality.

## Introduction

Intensive care units (ICUs) are specialized departments dedicated to the treatment of severely ill patients who require close monitoring and specific medical interventions to support vital functions. These units are equipped with advanced medical devices and demand a high level of attention. Patients with significantly compromised life functions find essential care in the ICU, where specialized treatment is administered. The ICU stands out from other hospital departments not only due to its technical sophistication but also its distinctive physical environment and unique treatment methodologies. The presence of a large number of personnel in the intensive care unit and the constant hum of medical devices may cause sensory overload in individuals [8, 48].

Consequently, patients in the ICU often contend with sensory deprivation, encountering challenges related to sensory input. To address these sensory issues in patients, it becomes crucial to implement psychosocial interventions alongside medical treatments. Psychological needs among nursing interventions for psychosocial needs identification and elimination, therapeutic relationship, interviewing, listening, empathy, giving information, and coping with stress practices such as training are included [3, 4]. Beyond the primary focus on medical care, these psychosocial approaches aim to enhance patient well-being and improve their overall quality of life. The implementation of such approaches holds the potential to mitigate physiological stress in patients, reducing the risk of complications associated with intensive care. These physiological values include physical parameters such as blood pressure, pulse, and pain [8, 9, 31, 42, 45, 48].

In the literature, it is highlighted that limitations in movement and body posture, coupled with social isolation, contribute to sensory deprivation in intensive care patients. This situation is associated with the development of ICU syndrome, characterized by symptoms such as diminished cognitive functions, restlessness, aggression, disruptions in the sleep–wake cycle, and disorientation [6, 45].

Nurses play a crucial role in addressing the sensory input problems of intensive care patients by incorporating psychosocial approaches into their care practices. These approaches, employed alongside medical treatments, aim to enhance patient care and improve the overall quality of life. Psychosocial interventions have demonstrated effectiveness in reducing physiological indicators like pulse, blood pressure, and respiration by activating sensory perceptions and inducing a relaxation effect and have contributed to the control and prevention of complications related to intensive care, such as sleep disturbances, pain, and anxiety [23, 44].

Expressive touch, music therapy, and aromatherapy are among the complementary treatment methods applied by nurses that can be applied for sensory input problems of intensive care patients and can positively affect sensory perceptions. Expressive touch, one of the planned sensory input applications, is touch with emotional content. With the touch movement applied to the skin, the receptors are stimulated and the messages to the brain are interpreted and responded to by the person. Touch makes the patient feel valued, increases patient-nurse communication, reduces the patient’s psycho-social problems, and affects physiological recovery by regulating respiration, blood pressure, and pulse [13, 23, 45].

Music therapy, which is also among the methods applied by nurses, lowers blood pressure, regulates the number of respirations, causes a decrease in pulse rate, and is used in patient care as a complementary method. Aromatherapy, which is one of the planned sensory input applications, is a complementary treatment method made with herbal essential oils. There are many oil extracts such as lemon balm, eucalyptus, and lavender used for aromatherapy. It is stated that these oils are effective in reducing pain, stress, and anxiety; improving stress coping mechanisms; and increasing the sense of psychological well-being [13, 14, 42].

A systematic review and meta-analysis focusing on the impact of family-centered sensory and emotional stimulation in comatose patients with traumatic brain injury revealed its effectiveness in improving the level of consciousness and cognition [49]. Another meta-analysis conducted by Liang et al. [26] found that nonpharmacological methods performed by nurses were effective in preventing delirium in individuals [26]. Li et al. [25] discovered in their meta-analysis that music therapy had a positive impact on the consciousness of individuals in intensive care units. This study aims to uncover the effectiveness of psychosocial interventions applied by nurses for addressing sensory deprivation in intensive care units.

## Materials and methods

This systematic review and meta-analysis study adhered to the PRISMA checklist (Preferred Reporting Items for Systematic Reviews and Meta-Analyses Protocols) guidelines [29] to ensure transparency and rigor. To minimize bias, the researchers (article author and non-author researcher) conducted the literature search, article selection, and data extraction with a dual scanning approach, which was then cross-verified by the researchers. The researchers (article author and non-author researcher) also personally performed the quality assessment of the studies included in the systematic review and meta-analysis.

### Inclusion and exclusion criteria

In this study, studies were screened according to PICOS:

- Study group (P: Patient): İntensive and critical care patients
- Intervention (I: Intervention): Psychosocial interventions
- Comparison (C: Comparison): No psychosocial interventions
- Outcomes (C: Outcomes): Sensory deprivation (level of consciousness, sleep disruption)
- Study design (S: Study design): Experimental, quasi-experimental studies published in Turkish and English

The exclusion criteria are as follows:

Letters to the editor and systematic and traditional review studies were excluded from the scope of this study.

### Search strategy

Between August 2023 and May 2024, a comprehensive search was executed across PubMed (including MEDLINE), EBSCO host Web of Science, Yök Thesis, CİNAHL, Embase, Cochrane Library, and Google Scholar using the MeSH-compatible keywords, such as (“critical care”[MeSH Terms] OR (“critical”[All Fields] AND “care”[All Fields]) OR “critical care”[All Fields]) AND (“nurses”[MeSH Terms] OR “nurses”[All Fields] OR “nurse”[All Fields]) AND (“psychosocial intervention”[MeSH Terms] OR (“psychosocial”[All Fields] AND “intervention”[All Fields]) OR “psychosocial intervention”[All Fields]). No temporal restrictions were imposed due to the limited number of studies on sensory deprivation, resulting in a scan of all available years.

### Selection of studies

**Fig. 1:**
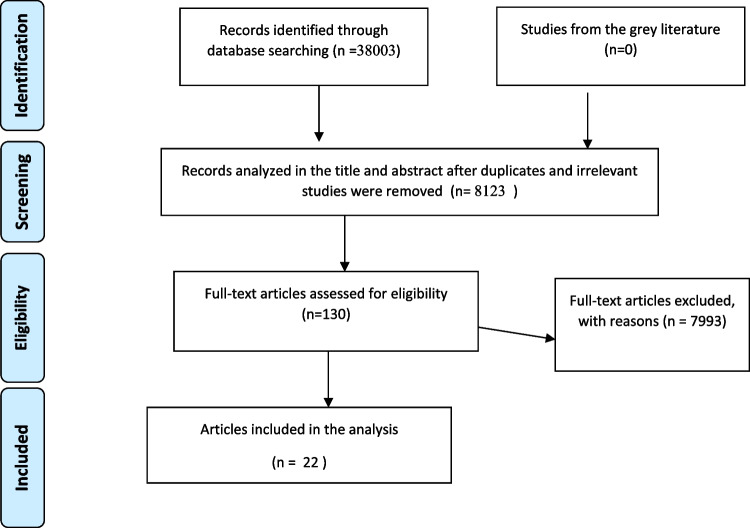
Selection of studies according to the PRISMA flow diagram

In the initial search, 38,003 records were identified. Following the removal of duplicate studies, 8123 records were analyzed based on their title and abstracts. After this initial review, 130 studies were chosen for a thorough examination in full text. Subsequently, these 130 full-text articles were assessed against predefined inclusion and exclusion criteria, leading to the inclusion of 22 studies that reported outcomes related to the impact of psychosocial interventions. Figure 1 provides a visual representation of the article selection process.

Potential articles were first screened by title and abstract. EndNote X8® (2019 Clarivate) was used to remove duplicates and organize the reference list.

### Data extraction

For data extraction, a tool developed by the researcher was employed to collect relevant information from the selected studies included in the systematic review meta-analysis. This tool facilitated the extraction of data on key aspects, such as author and publication year, study design, and the type of psychosocial intervention (Table 1).

**Table 1:** Characteristics and results of the included studies

Author/year	Patient group/intensive care unit	Study design	Origin of study	Sample size characteristics	Measurement tools	Psychosocial intervention applied	Duration of intervention	Main outcomes	Quality score
Salmani et al., 2017 [37]	Patients with brain injury/neurology intensive care	Randomized controlled	Iran	Experimental group: 30 Control group: 30	Coma Recovery Scale-Revised (CRS-R)	Family-centered emotional stimulation	2 weeks	Individuals in the experimental group received family-centered sensory stimulation. Individuals in the control group received routine emotional stimulation. Improvements in the level of consciousness were found to be more effective for those in the experimental group	Yes: 8/13 No: 3/13 Uncertain: 1/13 Not applicable: 1/13
Lee and Kang, 2020 [24]	Patients with cardiologic diseases/cardiology intensive care unit	Randomized controlled	South Korea	Experimental group: 24 Control group: 24	Pittsburgh Sleep Quality Index	Meditation through virtual reality	3 weeks	Individuals in the experimental group were meditated through virtual reality, and their sleep quality was found to be higher than the control group	Yes: 13/13
Naseri-Salahshou et al., 2018 [30]	Patients in the internal medicine intensive care unit	Randomized controlled	Iran	Experimental group: 33 Control group: 33	The Glasgow Coma Scale (GCS)	Listening to the Quran	1 week	Individuals in the experimental group were read suras from the Quran, and the level of consciousness of the individuals in the experimental group was found to be better	Yes: 13/13
Şirin Gök, 2015 [41]	Anesthesia intensive care	Quasi-experimental	Türkiye	43 patients	The Glasgow Coma Scale (GCS)	Listening to the voices of relatives	3 weeks	Individuals were made to listen to music, nature sounds, and the voices of their relatives, and it was found that the voices of their relatives positively affected the level of consciousness	Yes: 5/9 No: 3/9 Uncertain: 1/9
Öz and Cerit, 2023 [32]	Coronary intensive care unit	Randomized controlled	Türkiye	Experimental group: 23 Control group: 23	Richard–Campbell Sleep Questionnaire	Earplugs and eye patches	2 weeks	In the Coronary Intensive Care Unit, it was determined that eye patch and ear plugs had a positive effect on sleep quality in inpatients	Yes: 13/13
Karaman Özlü and Bilican, 2017 [33]	Surgical intensive care	Randomized controlled	Türkiye	Experimental group: 30 Control group: 30	The Richards-Campbell Sleep Scale	Aromatherapy massage	1 week	Individuals in the experimental group were massaged with aromatherapy, and significant improvements in sleep quality were observed	Yes: 13/13
Bahanor et al., 2019 [5]	Patients with traumatic brain injury/neurology intensive care	Randomized controlled	İran	Experimental group: 30 Control group: 30	The Glasgow Coma Scale (GCS)	Listening to nature sounds	2 weeks	Individuals in the experimental group were made to listen to nature sounds, and significant improvements were observed in the level of consciousness of the patients in the experimental group	Yes: 13/13
Sargolzaei et al., 2017 [39]	Intensive care patients with cerebrovascular disease	Randomized controlled	Iran	Experimental group: 40 Control group: 40	Sensory Modality Assessment and Rehabilitation Technique (SMART)	Sensory stimulation program	2 weeks	Sensory stimulation program was applied to the patients in the intervention group, and significant improvements were achieved in the sensory functions of the patients	Yes: 9/13 No: 2/13 Uncertain: 1/13 Not applicable: 1/13
Megha et al., 2013 [27]	Patients with traumatic brain injury/neurology intensive care	Randomized controlled	India	Experimental group: 10 Control group: 10	The Glasgow Coma Scale (GCS)	Multimodal coma stimulation	2 weeks	Multimodal coma stimulation was applied to the patients in the intervention group, and significant improvements were achieved in the level of consciousness of the patients	Yes: 8/13 No: 3/13 Uncertain: 1/13 Not applicable: 1/13
Çevik and Namık, 2018 [10]	Coma patients	Randomized controlled	Türkiye	Experimental group: 30 Control group: 30	The Glasgow Coma Scale (GCS)	Auditory stimuli	1 week	Auditory stimuli were applied to the patients in the intervention group, and significant improvements were achieved in the level of consciousness of the patients	Yes: 13/13
Urbenjaphol et al., 2009 [46]	Patients with traumatic brain injury/neurology intensive care	Randomized controlled	Thailand	Experimental group: 20 Control group: 20	The Glasgow Coma Scale (GCS)	Sensory stimulation program	2 weeks	A sensory stimulation program was applied to the patients in the intervention group, and significant improvements were achieved in the level of consciousness of the patients	Yes: 9/13 No: 2/13 Belirsiz: 1/13 Not applicable: 1/13
Henricson et al., 2008 [19]	General intensive care	Randomized controlled	Canada	Experimental group: 23 Control group: 23	APACHE, The Acute Physiology and Chronic Health Evaluation	Therapeutic touch	10 weeks	Relaxation was observed in individuals in the experimental group in which therapeutic touch was performed	Yes: 13/13
Uysal and Vaizoğlu, 2023 [47]	General intensive care	Quasi-experimental	Türkiye	135 patients	The Glasgow Coma Scale (GCS)	Interviewing family members through virtual reality	1 week	Video interviewing positively affected the coma scale scores of the patients	Yes: 5/9 No: 3/9 Uncertain: 1/9 Not applicable: 1/13
Moeini et al., 2010 [28]	Coronary intensive care	Randomized controlled	Iran	Experimental group: 32 Control group: 32	St. Mary’s Hospital Sleep Questionnaire (SMHSQ)	Aromatherapy	1 week	It was found that the sleep quality of the aromatherapy group was higher	Yes: 13/13
Rooin et al., 2023 [35]	Patients with traumatic brain injury/neurology intensive care	Randomized controlled	Iran	Experimental group: 45 Control group: 45	The Glasgow Coma Scale (GCS)	Reflexology-based foot massage	1 week	Individuals in the experimental group underwent reflexology and the level of consciousness of this group was found to be better	Yes: 13/13
Akpinar et al., [2]	General intensive care	Randomized controlled	Türkiye	Experimental group: 42 Control group: 42	Richards-Campbell Sleep Questionnaire (RCSQ)	Earplugs and eye patches	1 week	It was determined that eye patch and earplug application had a positive effect on sleep quality in patients hospitalized in the General Intensive Care Unit	Yes: 13/13
Şanlıtürk et al., 2023 [38]	COVID-19 intensive care	Randomized controlled	Türkiye	Experimental group: 43 Control group: 49	APACHE, The Acute Physiology and Chronic Health Evaluation	Cognitive Stimuli and Sleep Hygiene	4 weeks	Sensory stimulation and sleep hygiene interventions used in delirium prevention are effective in reducing the incidence of delirium in COVID-19 patients in intensive care	Yes: 9/13 No: 2/13 Uncertain: 1/13 Not applicable: 1/13
Parveen et al., [34]	Patients with traumatic brain injury/neurology intensive care	Randomized controlled	India	Experimental group: 40 Control group: 40	The Glasgow Coma Scale (GCS)	Auditory stimulation	2 weeks	Auditory stimulation by family members was found to be effective on the level of consciousness	Yes: 9/13 No: 2/13 Uncertain: 1/13 Not applicable: 1/13
Gorji et al., [16]	Patients with traumatic brain injury/neurology intensive care	Randomized controlled	Iran	Experimental group: 15 Control group: 15	The Glasgow Coma Scale (GCS)	Listening to the voices of relatives	2 weeks	Individuals in the experimental group were made to listen to the voices of their relatives and it was found that the level of consciousness of the individuals in the experimental group was better	Yes: 9/13 No: 2/13 Uncertain: 1/13 Not applicable:1/13
Karaman Özlü and Özer, 2017 [21]	Cardiovascular intensive care	Randomized controlled	Türkiye	Experimental group: 50 Control group: 50	Richards-Campbell Sleep Questionnaire (RCSQ)	Regulation of environmental factors	1 week	Environmental stimuli were reduced for the individuals in the experimental group and a significant increase in sleep quality was observed	Yes: 8/13 No: 3/13 Uncertain: 1/13 Not applicable: 1/13
Khojeh et al., 2018 [22]	General intensive care	Randomized controlled	İran	Experimental group: 20 Control group: 20	The Glasgow Coma Scale (GCS)	Listening to the voices of relatives	1 week	Individuals in the experimental group were made to listen to the voices of their relatives and it was found that the level of consciousness of the individuals in the experimental group was better	Yes: 9/13 No: 2/13 Uncertain: 1/13 Not applicable: 1/13
Chuaykarn et al., 2017 [11]	Patients with traumatic brain injury/neurology intensive care	Randomized controlled	Thailand	Experimental group: 15 Control group: 15	Coma Recovery Scale-Revised (CRS-R)	Sensory stimulation program	2 weeks	Sensory stimulation program was applied to the patients in the intervention group and significant improvements were observed in the level of consciousness of the patients	Yes: 13/13

### Research ethics

This study is a systematic review and meta-analysis and is based on published studies.

### Assessment of the methodological quality of studies

The quality assessment of the studies included in this systematic review and meta-analysis was conducted using quality assessment tools prepared based on the study design by The Joanna Briggs Institute (The Joanna Briggs Institute Critical Appraisal Tools for Use in IBI Systematic Reviews, 2021). The tool contains information on sample representativeness of the target population, participant recruitment, adequacy of the sample size, detailed description of the study subjects and study setting, sufficient coverage of the data analysis, objective criteria in the measurement of the outcome variable, and identification of subpopulation, reliability, appropriate statistical analysis, and identification of confounding variables. The quality scores of the included studies were assessed and presented using the mean scores to designate them as high or low quality. The JBI tool for prevalence studies was used as a guideline for data extraction from the selected articles.

The selection of evaluation tools in this study was tailored to the specific designs of the included studies in the systematic review and meta-analysis. For randomized controlled trials, a set of 13 questions was employed (The Joanna Briggs Institute Critical Appraisal Tools for Use in IBI Systematic Reviews, 2021), while quasi-experimental studies were evaluated using 9 questions [43]. Responses to these questions were “Yes, No, Uncertain, Not Applicable.” The methodological quality of the studies was categorized as “mediocre” if fewer than 50% of items were rated as “yes,” “moderate” if 51–80% of items were rated as “yes,” and “good” if more than 80% of items were rated as “yes.”

The data extraction tool included crucial information such as the author and year of the study, title, year of study, year of publication, study area and country, sub-region, study design and type, study population, age of participants, sample size, response rate, and the measured outcome. The evaluation results for each study are presented in Table 1 as “Quality Score.”

### Data synthesis

This study utilized CMA Ver. 2 for conducting statistical calculations. The assessment of heterogeneity among the studies involved the application of Cochrane Q and Higgins *I*^2^ tests. A Higgins *I*^2^ value exceeding 50% was considered indicative of significant heterogeneity. SMD (standardized mean difference) was calculated at a 95% confidence interval (CI) for each outcome variable in the study. Statistical significance was considered at *p* < 0.05 for all tests.

Moderator analysis and sensitivity analysis were conducted to explore the sources of heterogeneity. Funnel plots, Egger test, and Begg and Mazumdar Rank Correlation analyses were conducted to evaluate publication bias.

## Results

Twenty of the studies included in the study were randomized-controlled experimental studies and two were quasi-experimental with a pre-post-test and a control group. The total sample size of the studies was 1294 (intervention group = 555; control group = 561; single group = 178) (Table 1).

The systematic review and meta-analysis included studies that collectively demonstrated that over 50% of the criteria in the assessment tool were met (Table 1); this indicates a moderate quality of evidence. This underscores the reliability of the analysis findings, which draw from studies with commendable evidence quality.

### Meta-analysis results on the effectiveness of nurses’ psychosocial interventions for sensory deprivation in intensive care units on the level of consciousness of individuals

**Fig. 2:**
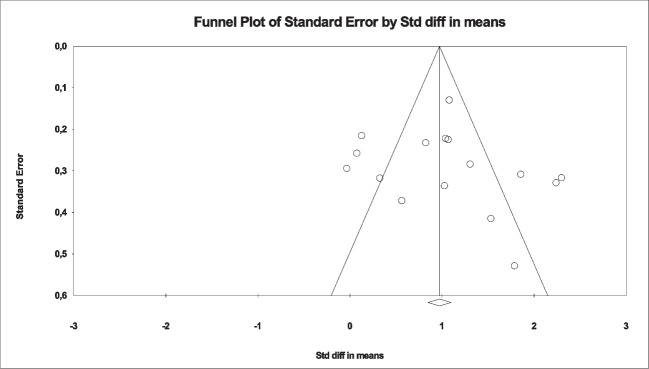
Funnel plot of the level of consciousness

Due to the considerable heterogeneity among the studies, a random effects model was employed (*I*^2^ = 83.64%; *Q* = 91,711; *p* > 0.05). The systematic review and meta-analysis results revealed the effectiveness of nurses’ psychosocial interventions for sensory deprivation in intensive care units, demonstrating significant enhancements, particularly in patients’ level of consciousness (SMD = 1.042, %95 CI = 0.716 to 1.369; *Z* = 6.25; *p* < 0.05). Sensitivity analysis, conducted using Egger and Begg tests, indicated no evidence of publication bias (Egger test: *t* = 0.69, *p* = 0.49; Begg test: *z* = 1.30, *p* = 0.19). Additionally, the absence of publication bias was confirmed through an examination of the funnel plot (see Fig. 2).

**Fig. 3:**
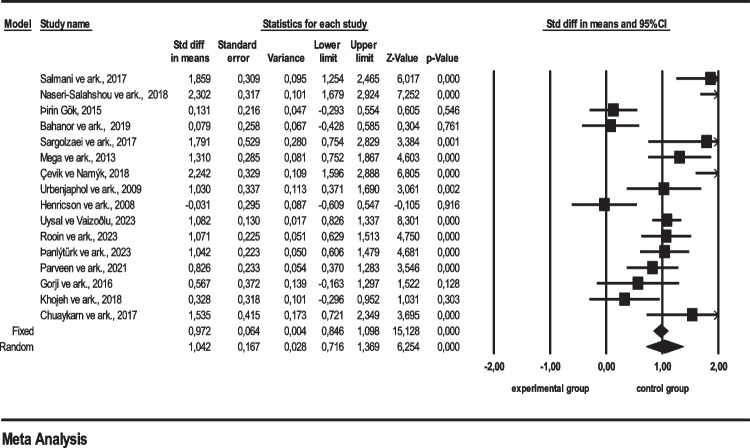
Forest plot of the level of consciousness

The effect sizes, standardized as Cohen’s *d* or Hedges’s *g*, were utilized to measure the effect size [17]. In this investigation, Cohen’s *d* was employed, with statistical significance set at 95%. Regarding the effect sizes, Cohen’s [12] criteria were referenced, where values falling between 0.75 and 1.10 indicate a large effect. An average effect size of ES = 1.042 was calculated (Table 2). Based on these calculations, data from 16 studies included in the systematic review meta-analysis suggest that psychosocial interventions by nurses for the level of consciousness in intensive care units yield a significant impact on individuals according to the random effects model [12]. The forest plot of these 16 studies is provided below in Fig. 3.

**Table 2:** Moderator results for the effect of nurses’ psychosocial interventions for sensory deprivation in intensive care units

Moderator	Number of studies	Effect size	Standard error	Lower limit	Upper limit	*p*
Type of intensive care unit						
Neurology intensive care	9	1.072	0.191	0.698	1.447	**0.000**
General intensive care	6	1.025	0.342	0.354	1.696	**0.003**
Cardiology intensive care	3	0.517	0.444	− 0.354	1.388	**0.245**
Anesthesia intensive care	1	0.131	0.216	− 0.293	0.554	0.546
Surgical intensive care	1	2.133	0.323	1.500	2.676	**0.000**
COVID-19 intensive care	1	1.042	1.223	0.606	1.479	**0.000**
Cardiovascular surgery intensive care	1	2.130	0.250	1.639	2.621	**0.000**
Total	22	0.961	0.055	0.852	1.069	**0.000**
Type of psychosocial intervention implemented						
Listening to the voices of relatives	4	0.713	0.403	− 0.077	0.502	0.077
Sensory stimulation program	3	1.340	0.234	0.881	1.800	**0.000**
Providing auditory stimuli	2	1.515	0.709	0.128	2.902	**0.032**
Earplugs and eye patches	2	0.848	0.566	− 0.262	1.958	0.134
Multimodal coma stimulation	1	1.310	0.285	0.752	1.867	**0.000**
Aromatherapy	1	0.352	0.252	− 0.142	0.846	0.162
Reflexology-based foot massage	1	1.070	0.225	0.629	1.513	**0.000**
Cognitive stimulus and sleep hygiene training	1	1.042	0.233	1.606	1.479	**0.000**
Regulation of environmental factors	1	2.130	0.250	1.639	2.621	**0.000**
Interviewing family members through virtual reality	1	− 0.199	0.084	− 0.766	0.368	0.492
Aromatherapy massage	1	2.133	0.323	1.500	2.676	**0.000**
Listening to nature sounds	1	0.079	0.258	− 0.428	0.585	0.761
Listening to the Quran	1	2.302	0.317	1.679	2.924	**0.000**
Meditation through virtual reality	1	1.082	0.130	0.826	1.337	**0.000**
Therapeutic touch	1	− 0.031	0.295	− 0.609	0.547	0.916
Total	22	1.032	0.066	0.903	1.162	**0.000**
Duration of psychosocial intervention						
1 week	8	1.211	0.266	− 0.689	1.732	**0.000**
2 weeks	8	1.081	0.226	0.637	1.525	**0.000**
3 days	2	0.013	0.173	− 0.992	− 0.001	0.914
1 day	1	2.133	0.323	− 0.326	0.352	**0.000**
10 weeks	1	− 0.031	0.295	− 0.609	0.547	0.946
2 days	1	1.442	0.331	0.793	2.090	**0.000**
4 weeks	1	1.042	0.223	0.606	1.479	**0.000**
Total	22	0.791	0.092	0.610	0.972	**0.000**
Patient group						
Patients with traumatic brain injury	8	1.016	0.197	0.630	1.402	**0.000**
Patients with internal diseases	6	1.025	0.342	0.354	1.696	**0.003**
Patients with cardiological diseases	4	0.931	0.543	− 0.132	− 1.995	0.086
Patients who have undergone surgical operations	1	2.133	0.323	− 1.500	2.676	**0.000**
Patients with COVID-19 disease	1	1.042	0.223	0.606	1.479	**0.000**
Patients with neurological and metabolic diseases	1	0.131	0.216	− 0.293	0.554	0.546
Patients with cerebrovascular disease	1	1.791	0.529	0.754	2.829	**0.001**
Total	22	0.959	0.104	0.756	1.163	**0.000**
Age range of the sample group						
18–65 years old	15	0.935	0.201	0.541	1.330	**0.000**
15–65 years old	1	0.826	0.233	0.370	1.283	0.303
18–50 years old	1	0.328	0.318	− 0.296	1.031	0.303
18–60 years old	1	1.535	0.415	0.721	2.349	**0.000**
20–60 years old	1	2.302	0.317	1.679	2.924	**0.000**
25–55 years old	1	1.310	0.285	0.752	1.867	**0.000**
35–75 years old	1	1.791	0.529	0.754	2.829	**0.001**
45–65 years old	1	1.042	0.233	0.606	1.479	**0.000**
Total	22	1.115	0.980	0.924	1.307	**0.000**

### Meta-analysis results on the effect of nurses’ psychosocial interventions for sensory deprivation on sleep quality in intensive care units

**Fig. 4:**
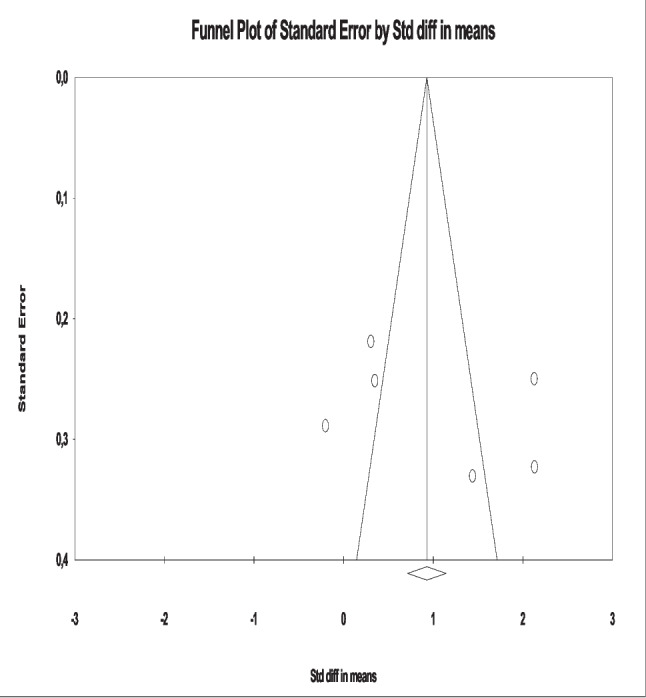
Funnel plot of sleep quality variable

Since these studies had a high level of heterogeneity, random effects model was used (*I*^2^ = 92.61%; *Q* = 67.72, *p* > 0.05). The result of the meta-analysis shows that psychosocial interventions by nurses for sensory deprivation in intensive care units are effective on patients and provide significant improvements in sleep quality (SMD=1.21, 95% CI= 0.232 to 1.810; *Z* = 2.49; *p* < 0.05). Egger and Begg tests were used for sensitivity analysis (Egger test: *t* = 0.78, *p* = 0.47) and no publication bias was found (Begg test: *z* = 0.00, *p* = 1.00). In addition, no publication bias was found in the funnel plot (Fig. 4).

**Fig. 5:**
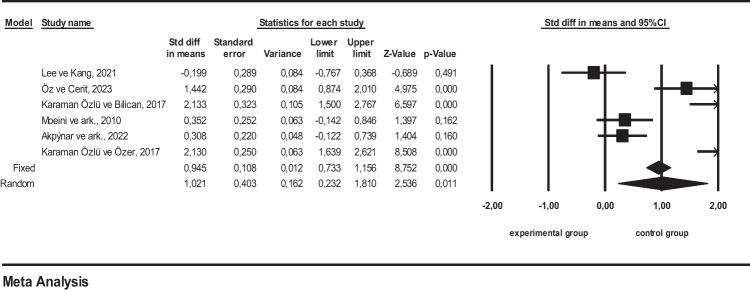
Forest plot of sleep quality variable

While interpreting the effect sizes, the effect size classification determined by Cohen [12] was taken into consideration and the value between 0.75 and 1.10 indicates a large effect. The average effect size value ES = 1.21 was calculated (Table 2). The data in the 6 studies included in the meta-analysis in line with the calculations show that psychosocial interventions by nurses for sensory deprivation in intensive care units have a large effect on the sleep quality of individuals according to the random effects model [12]. The forest plot of the 6 studies within the scope of the research is given in Fig. 5.

### Moderator results for the effect of nurses’ psychosocial interventions for sensory deprivation in intensive care units

Moderator analyses were conducted for the effect of psychosocial interventions made by nurses for sensory deprivation in intensive care units. Moderator evaluations include subgroup and meta-regression analyses. Moderator analyses performed in systematic reviews and meta-analyses are used to test which factors (moderators) can explain the differences in the observed effect size [18].

In the study, moderator analyses were performed for the type of intensive care unit in which the patient stayed, the type of psychosocial intervention applied, the duration of the psychotherapeutic intervention applied, and the age range of the sample group.

The average effect size for the specific type of intensive care unit where patients were hospitalized was determined to be 0.961 with a significance level of *p* < 0.05. This study revealed that the type of intensive care unit had an impact on the size of the effect. Additionally, the average effect size for the type of psychosocial intervention utilized in the systematic review meta-analysis was calculated at 1.032 with a significance level of *p* < 0.05.

It was observed that the type of psychosocial intervention influenced the size of the effect of psychotherapeutic interventions administered to individuals. Through moderator analysis, it was established that the duration of psychotherapeutic intervention (SMD = 0.791 and *p* < 0.05), patient group (SMD = 0.959 and *p* < 0.05) and age range of the sample group (SMD = 1.115 and *p* < 0.05) all played significant roles in determining the effect size of psychosocial interventions (Table 2).

## Discussion

This study concludes that nurses’ psychosocial interventions for sensory deprivation in intensive care units effectively improved patients’ levels of consciousness and sleep quality. In line with our findings, a systematic review and meta-analysis by Hwang and Shin [20] revealed that nurses’ use of aromatherapy in intensive care units prevented sleep disruption and notably enhanced sleep quality [20]. Similarly, Li et al. [25] conducted a meta-analysis demonstrating that music therapy positively impacted individuals’ consciousness states in intensive care units [25]. Another systematic review conducted by Sahawneh and Boss [36] revealed that nonpharmacological methods implemented by nurses effectively reduced both the occurrence and duration of delirium [36]. These findings underscore the pivotal role of psychosocial methods employed by nurses in addressing sensory deprivation among individuals in intensive care settings.

Furthermore, the study identified a significant correlation between the type of psychosocial intervention utilized and the effectiveness of interventions on individuals. Likewise, a systematic review led by Bellon et al. [7] indicated that interventions such as earplugs, eye masks, music, and acupuncture were effective and led to notable enhancements in individuals’ sleep quality [7]. Similarly, Abbas et al. [1] reported in their systematic review that earplugs and aromatherapy massage played crucial roles in reducing sleep disruption. Earplugs and aromatherapy massage were also reported to alleviate anxiety and enhance sleep quality among individuals [40]. In our study, various interventions, including sensory stimulation programs, auditory stimulation, multimodal coma stimulation, reflexology-based foot massage, cognitive stimulation, sleep hygiene training, regulation of environmental factors, and aromatherapy, were found to significantly improve individuals’ level of consciousness and sleep quality. These findings reinforce the multifaceted approaches available to nurses for addressing sensory deprivation and enhancing the well-being of patients in intensive care units.

The study identified that the type of intensive care unit where patients were hospitalized significantly influenced the effectiveness of psychosocial interventions. In our investigation, psychosocial interventions administered to patients across various intensive care units, including neurology, general, surgical, COVID-19, and cardiovascular surgery intensive care, were found to yield positive outcomes. Notably, a systematic review and meta-analysis focusing on family-centered sensory and emotional stimulation in neurology intensive care patients with traumatic brain injury highlighted its effectiveness in enhancing patients’ level of consciousness and cognition [49]. This suggests that regardless of the specific intensive care unit, the intensive care environment can detrimentally impact individuals, emphasizing the importance of psychosocial interventions.

Moreover, our study revealed that the age range of the sample group played a crucial role in determining the effectiveness of psychosocial interventions in intensive care units. Specifically, interventions applied to individuals aged 18 and older were found to effectively alleviate sensory deprivation. A meta-analysis conducted by Erwin et al. [15] underscored the presence of risk factors for delirium and sensory deprivation in pediatric intensive care units. Notably, the use of physical restraint and mechanical ventilation in children was identified as particularly detrimental [15]. These findings highlight the importance of tailoring psychosocial interventions to the unique needs and vulnerabilities of patients across different age groups in intensive care settings.

The duration of psychosocial interventions emerged as a significant factor influencing their effectiveness in our study. Specifically, interventions lasting 1, 2, and 4 weeks, as well as those administered over 1 or 2 days, were found to be effective for individuals in intensive care units. Conversely, contrasting results were observed in a study focusing on neurology intensive care patients with traumatic brain injury, where the duration of intervention did not impact the effect size [49]. Discrepancies between our findings and those in the literature may stem from differences in intervention procedures and inclusion criteria across studies.

## Conclusion and recommendations

This study highlights the effectiveness of nurses’ psychosocial interventions for alleviating sensory deprivation in intensive care units, leading to significant enhancements in patients’ levels of consciousness and sleep quality. Various factors, including the type of psychosocial intervention, the age range of the sample, the type of intensive care unit, the patient group, and the duration of the intervention, were identified as influential in the effectiveness of psychosocial interventions. Specifically, interventions such as sensory stimulation programs, auditory stimulation, multimodal coma stimulation, reflexology-based foot massage, cognitive stimulation, sleep hygiene training, regulation of environmental factors, and aromatherapy significantly improved individuals’ level of consciousness. It is thought that the transformation and application of intensive care nurses’ knowledge about psychosocial care into skills in providing holistic care to patients receiving treatment in intensive care will contribute to increasing the quality of nursing care. In this context, it is recommended that nursing interventions with proven effectiveness through evidence-based research on sensory deprivation problems in intensive care patients should be used in patient care and nurses should be supported in this regard.

## Relevance for clinical practice

Based on the findings of this study, psychosocial interventions by nurses to prevent and reduce sensory deprivation can be used to positively affect the level of consciousness and sleep quality of individuals in intensive care.

## Limitation of studies

The presence of small sample sizes, lack of blinding, and utilization of pre- and post-test designs in some of the studies included in the analysis may be viewed as limitations of this study, which could potentially diminish the robustness of the evidence supporting the study’s findings.

## Data Availability

The datasets used and/or analyzed during the current study are available from the corresponding author on reasonable request.
